# n^+^ GaAs/AuGeNi-Au Thermocouple-Type RF MEMS Power Sensors Based on Dual Thermal Flow Paths in GaAs MMIC

**DOI:** 10.3390/s17061426

**Published:** 2017-06-17

**Authors:** Zhiqiang Zhang, Xiaoping Liao

**Affiliations:** Key Laboratory of MEMS of the Ministry of Education, Southeast University, Nanjing 210096, China; xpliao@seu.edu.cn

**Keywords:** n^+^ GaAs/AuGeNi-Au thermocouples, thermal flow path, GaAs MMIC, microwave measurement, power sensor, RF MEMS

## Abstract

To achieve radio frequency (RF) power detection, gain control, and circuit protection, this paper presents n^+^ GaAs/AuGeNi-Au thermocouple-type RF microelectromechanical system (MEMS) power sensors based on dual thermal flow paths. The sensors utilize a conversion principle of RF power-heat-voltage, where a thermovoltage is obtained as the RF power changes. To improve the heat transfer efficiency and the sensitivity, structures of two heat conduction paths are designed: one in which a thermal slug of Au is placed between two load resistors and hot junctions of the thermocouples, and one in which a back cavity is fabricated by the MEMS technology to form a substrate membrane underneath the resistors and the hot junctions. The improved sensors were fabricated by a GaAs monolithic microwave integrated circuit (MMIC) process. Experiments show that these sensors have reflection losses of less than −17 dB up to 12 GHz. At 1, 5, and 10 GHz, measured sensitivities are about 63.45, 53.97, and 44.14 µV/mW for the sensor with the thermal slug, and about 111.03, 94.79, and 79.04 µV/mW for the sensor with the thermal slug and the back cavity, respectively.

## 1. Introduction

Radio frequency (RF) power measurement plays an important role in radar and communication systems. Thermocouple-type RF power sensors based on the Seebeck effect have become one of the most widely tools for RF power detection, gain control, and circuit protection [1]. They show good linearity, compared to diode-type and thermistor-type sensors, as well as zero dc power consumption, among other advantages [2]. However, the sensors that are fabricated using traditional Si and GaAs processes suffer from low sensitivity due to the thermal loss of the substrate. Recently, some thermocouple-type RF power sensors based on the microelectromechanical system (MEMS) technology have been developed [3,4,5,6,7,8]. To increase the sensitivity, they focus on studying bulk-etching substrate techniques, such as front-etching [3,4] and back-etching [5,6,7,8]. The thinner the etched substrate is, the higher the sensitivity is. However, only the method of thinning the substrate is used to increase the heat transfer efficiency. Therefore, a very or extreme thin substrate membrane (~1 µm) is usually designed, which bring challenges to the reliability and packaging of the devices.

In order to solve the above problem, n^+^ GaAs/AuGeNi-Au thermocouple-type RF MEMS power sensors based on dual thermal flow paths are proposed in this paper. In the design, a suitable thermal slug and an appropriate substrate membrane structure as upper and lower heat conduction paths are researched to improve the efficiency of the heat transfer. In the fabrication, the substrate membrane of the sensors is about 10 µm thick to obtain a low thermal loss of the substrate and a sufficiently robust stiffness, compatible with the GaAs monolithic microwave integrated circuit (MMIC) process. These sensors can be easily packaged by using the bonding or soldering methods [9]. The purpose of this work is to achieve acceptable sensitivity with good reliability.

## 2. Principle and Design

**Figure 1:**
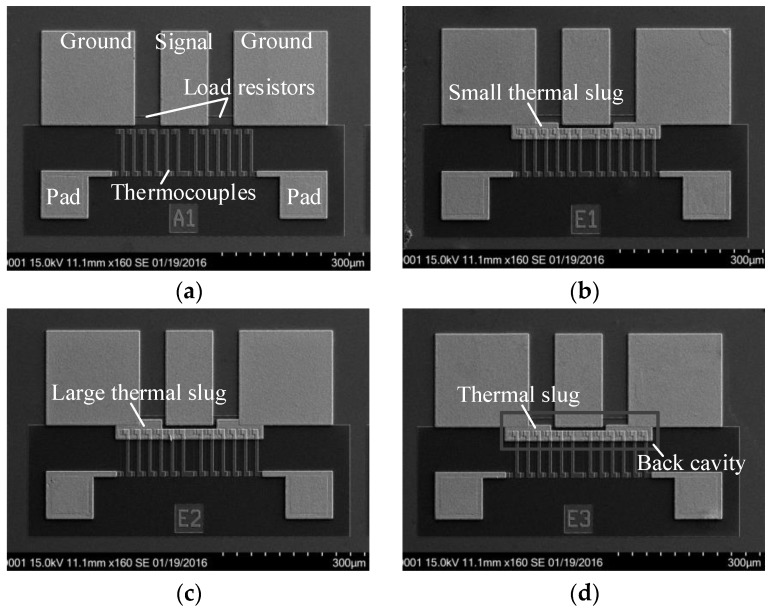
SEM views of four n^+^ GaAs/AuGeNi-Au thermocouple-type RF MEMS power sensors based on GaAs MMIC process. (**a**) Sensor A1 with a basic structure. (**b**) Sensor E1 with a small thermal slug. (**c**) Sensor E2 with a large thermal slug. (**d**) Sensor E3 with a thermal slug and a back cavity (dual thermal flow paths).

Figure 1 shows four n^+^ GaAs/AuGeNi-Au thermocouple-type RF MEMS power sensors. Each sensor includes a CPW transmission line, two load resistors, 16 thermocouples, and two output pads. The characteristic impedance of the CPW is designed to be 50 Ω. The two resistors are connected to the CPW in parallel, where the resistance of each resistor is designed to be 100 Ω. This means that the parallel resistors match the CPW. The thermocouples are placed near the resistors. The ends of the thermocouples in proximity to and away from the resistors are called the hot and cold junctions, respectively. The distance of the resistors and the thermocouples is designed to be 10 µm. The principle of the sensors is that, when the RF power is input on the CPW, the load resistors dissipate the power and generate heat; following that, the resulting heat is transferred to the hot junctions of the thermocouples; finally, the thermocouples convert the heat into an output thermovoltage based on the Seebeck effect. The model of the thermocouple-type RF power sensors has been established to describe the temperature distribution [10,11].

In this paper, the sensor A1 is given as a traditional basic structure for comparison, the sensors E1 and E2 are improved structures with small and large thermal slugs, respectively, and the sensor E3 is an improved structure with the thermal slug and a back cavity. In the design of the sensors E1 and E2, a thermal slug with different lengths is placed between the resistors and the thermocouples. The thermal slug is made of a thick gold. The method is able to increase the efficiency of heat transfer by adding a heat conduction path above the substrate. It should be noted that the overlapping lengths between the slug and the resistors are 2 and 10 µm for the sensors E1 and E2, respectively. In order to sufficiently absorb heat around the heating resistors, the slugs are connected to ground lines of the CPW. In the design of the sensor E3, besides the thermal slug, a back cavity with a robust stiffness of the substrate membrane is fabricated underneath the resistors and the hot junctions of the thermocouples. After taking the reliability and packaging into account, the thickness of the membrane is designed to be 10 µm. The method obviously reduces the thermal loss of the substrate and improves the efficiency of heat transfer. It is equivalent to add a heat conduction path of the substrate. Therefore, the sensor E3 offers two thermal flow paths to increase the sensitivity.

## 3. Fabrication

**Figure 2:**
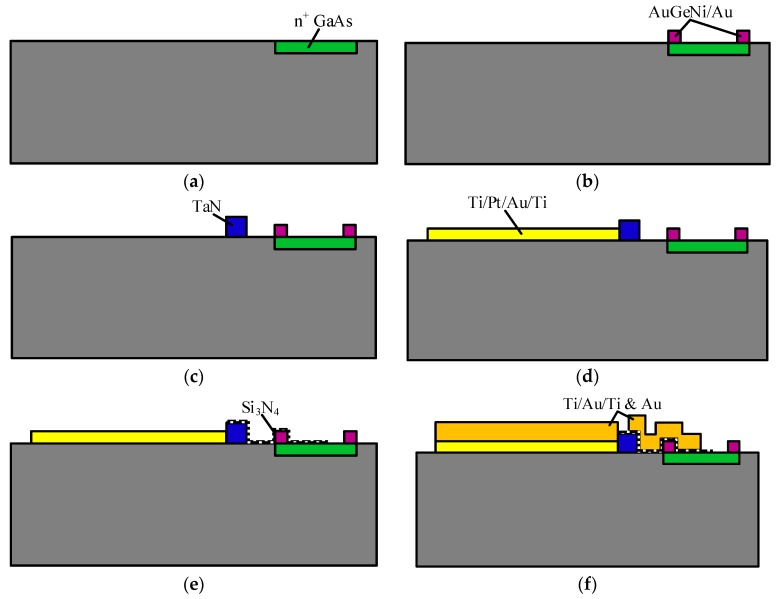

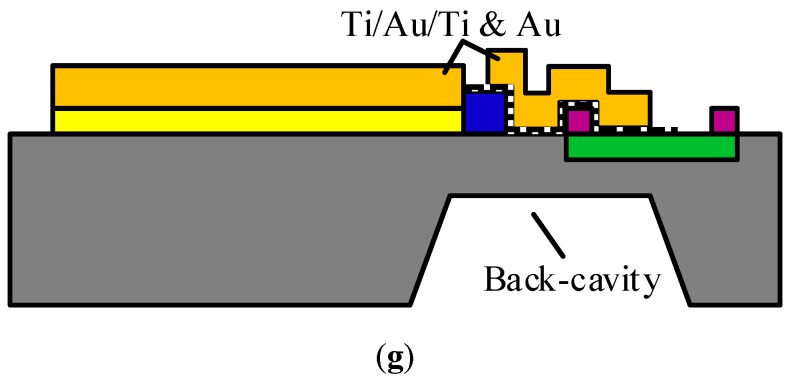
Fabrication process of the thermocouple-type RF MEMS power sensors with a thermal slug and a back cavity (dual thermal flow paths). (**a**) n^+^ GaAs; (**b**) AuGeNi/Au; (**c**) TaN; (**d**) Ti/Pt/Au/Ti; (**e**) Si_3_N_4_; (**f**) Ti/Au/Ti &Au; (**g**) Back-cavity.

These n^+^ GaAs/AuGeNi-Au thermocouple-type RF MEMS power sensors based on dual thermal flow paths are fabricated using the GaAs MMIC process [12]. (a) The GaAs substrate with an epitaxial layer of n^+^ GaAs is chosen, where the doping concentration of n^+^ GaAs is 1.0 × 10^18^ cm^−3^ for fabricating ohmic contact places. The epitaxial layer is etched to be about 1.0 × 10^17^ cm^−3^ for fabricating semiconductor arms of the thermocouples. (b) A metal layer of AuGeNi-Au is sputtered for fabricating metal arms of the thermocouples and patterned using a liftoff process. c) A layer of TaN is sputtered to fabricate the load resistors and patterned by a liftoff process. The square resistance of TaN is 25 Ω/□. (d) A layer of Ti/Pt/Au/Ti is evaporated to form the CPW and output pads and patterned by a liftoff process. The thickness of the Ti/Pt/Au/Ti layer is 500/300/3500/500 Å. (e) A dielectric layer of Si_3_N_4_ is deposited by plasma enhanced chemical vapor deposition (PECVD) and patterned to isolate the thermal slug from the load resistors so as to avoid shorting. The thickness of the dielectric layer is 0.1 μm. (f) A seed layer of Ti/Au/Ti is evaporated and patterned. After removing the top layer of Ti, a metal layer of Au is electroplated for thickening the CPW and pads and fabricating the thermal slug. The thickness of the metal layer is about 2 μm. (g) The substrate of GaAs is thinned, and the back cavity is fabricated to form the substrate membrane underneath the resistors and the hot junctions of the thermocouples using a via-hole etching technique. The thickness of the membrane is about 10 µm. Figure 2 shows the fabrication process of the thermocouple-type RF MEMS power sensors with a thermal slug and a back cavity (dual thermal flow paths).

## 4. Measurement and Analysis

**Figure 3:**
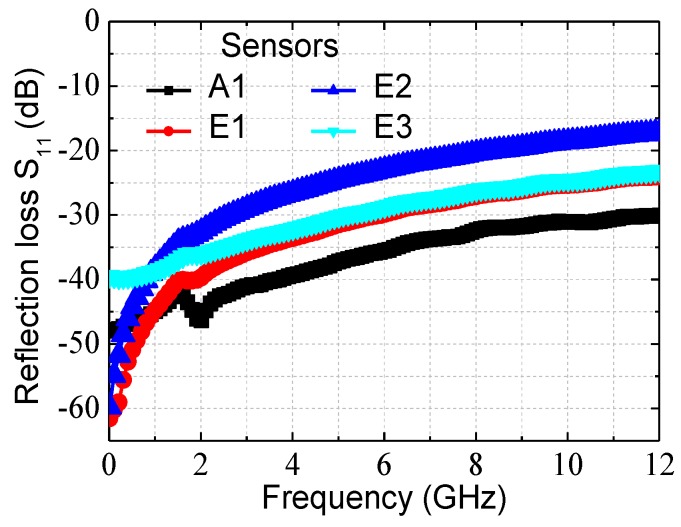
Measured reflection loss S_11_ of these n^+^ GaAs/AuGeNi-Au thermocouple-type RF MEMS power sensors.

Reflection losses of the four n^+^ GaAs/AuGeNi-Au thermocouple-type RF MEMS power sensors were measured using an Agilent N5244A network analyzer and a Cascade RF probe station. Figure 3 shows measured reflection losses of the four RF power sensors. To obtain an accurate RF measurement, the network analyzer is calibrated using a short-open-load-thru calibration technique. The measured S_11_ are less than −24.5 dB below 5 GHz, −18.6 dB below 10 GHz, and −17.1 dB up to 12 GHz. These results mean that they have the good RF impedance matching between the CPW and the resistors. In Figure 3, the reflection losses of the sensors E1 and E3 overlap almost completely in the high frequency range, but show some differences the low frequency of 0.01–2 GHz. For the sensors E1 and E3, the S_11_ are −44.7 (0.0034%) and −38.7 dB (0.0135%) at 1 GHz, and −39.7 (0.0107%) and 36.0 dB (0.0251%) at 2 GHz, respectively. They are very small values, and the differences can result from different load resistance after the fabrication and dielectric losses and parasitics of the substrate. As can be seen, the sensor E2 are higher S_11_ than others at 2–12 GHz, but are acceptable for RF applications compared to [13]. In order to prevent the electromagnetic coupling loss between the resistors and thermocouples, the thermal slug is connected with a ground line of the CPW. Such design shows that the thermal slug will slightly reduce the resistance of the load resistors, which leads to the impedance mismatching between the resistors and the CPW. Therefore, the large thermal slug causes a greater reduction in load resistance than the small slug. In addition, the impedance mismatching leads to the fact that the incident power is reflected to a greater extent in the input port, which results in less sensitivity.

**Figure 4:**
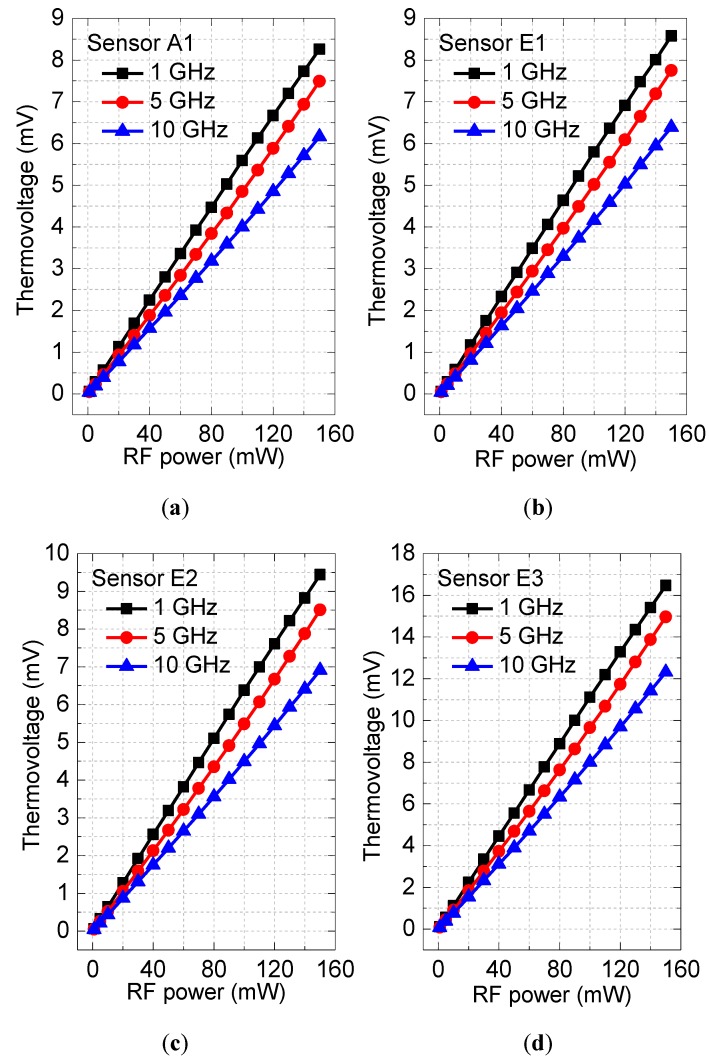
Measured output thermovoltages with respect to input RF power at 1, 5, and 10 GHz of these RF power sensors. (**a**) The sensor A1, (**b**) the sensor E1, (**c**) the sensor E2, and (**d**) the sensor E3.

Sensing performances of the four n^+^ GaAs/AuGeNi-Au thermocouple-type RF MEMS power sensors were measured using an Agilent E8257D PSG analog signal generator, a Cascade RF probe station, and a Fluke 8808A digital multimeter. The signal generator was used to generate RF signals with different power and frequency, and the multimeter was used to record the output thermovoltages. Figure 4 shows measured output thermovoltages with respect to the input RF power for the four RF power sensors. It was found by the measurement that the thermovoltages increase with the increase in the RF power, and the relationships between the input and output show good linearity. When the RF power is 150 mW for the sensors A1, E1, E2, and E3, the thermovoltages are about 8.26, 8.58, 9.44, and 16.47 mV at 1 GHz, 7.49, 7.75, 8.51, and 14.96 mV at 5 GHz, and 6.16, 6.39, 6.91, and 12.31 mV at 10 GHz, respectively. Table 1 shows the average sensitivities of the four n^+^ GaAs/AuGeNi-Au thermocouple-type RF power sensors. In Table 1, the sensors E1 and E2 show larger sensitivities than the sensor A1, which verifies the design validity of the thermal slug. In addition, the sensor E2 with the large thermal slug produces larger outputs than the sensor E1. Such results show that the large overlapping length is better for increasing the efficiency of heat transfer. Finally, the experiments demonstrate that the sensor E3 with two thermal flow paths results in maximum sensitivity, which is consistent with the design.

## 5. Conclusions

In this paper, n^+^ GaAs/AuGeNi-Au thermocouple-type RF MEMS power sensors are proposed based on dual thermal flow paths of the thermal slug and the back cavity. The different overlapping of the thermal slug was investigated. The sensors result in good sensitivity, with a sufficiently robust stiffness membrane. This design can become an important reference for research on other thermocouple-type sensors.

## Figures and Tables

**Table 1:** Comparison of average sensitivities of the four n^+^ GaAs/AuGeNi-Au thermocouple-type RF power sensors in GaAs MMIC.

Sensors	Sensitivities (µV/mW)		
	1 GHz	5 GHz	10 GHz
A1	56.28	47.97	39.68
E1	58.05	49.59	41.16
E2	63.45	53.97	44.14
E3	111.03	94.79	79.04
